# Monitoring neoadjuvant therapy responses in rectal cancer using multimodal nonlinear optical microscopy

**DOI:** 10.18632/oncotarget.22366

**Published:** 2017-11-03

**Authors:** Lian-Huang Li, Zhi-Fen Chen, Xing-Fu Wang, Xing Liu, Wei-Zhong Jiang, Shuang-Mu Zhuo, Li-Wei Jiang, Guo-Xian Guan, Jian-Xin Chen

**Affiliations:** ^1^ Key Laboratory of OptoElectronic Science and Technology for Medicine of Ministry of Education, Fujian Provincial Key Laboratory for Photonics Technology, Fujian Normal University, Fuzhou, Fujian, China; ^2^ Department of Colorectal Surgery, Fujian Medical University Union Hospital, Fuzhou, Fujian, China; ^3^ Department of Pathology, The First Affiliated Hospital of Fujian Medical University, Fuzhou, Fujian, China

**Keywords:** neoadjuvant therapy, rectal cancer, tumor response, stromal response, nonlinear microscopy

## Abstract

Most patients with rectal cancer have a better prognosis after receiving neoadjuvant therapy because of its remarkable curative effect. However, no device delivers real-time histopathologic information on treatment response to help clinicians tailor individual therapeutic strategies. We assessed the potential of multimodal nonlinear optical microscopy to monitor therapeutic responses, including tumoral and stromal responses. We found that two-photon excited fluorescence imaging can, without labeling, identify colloid response, inflammatory cell infiltration, vascular proliferation, and tumor regression. It can also directly detect rare residual tumor cells, which may be helpful for distinguishing tumor shrinkage from tumor fragmentation. In addition, second harmonic generation imaging shows the ability to monitor three types of fibrotic responses: mature, intermediate, and immature. We also determined nonlinear spectra, collagen density, and collagen orientation indexes to quantitatively analyze the histopathologic changes induced by neoadjuvant therapy in rectal cancer. Our work demonstrates that nonlinear optical microscopy has the potential to become a label-free, real-time, *in vivo* medical imaging technique and provides the groundwork for further exploration into the application of nonlinear optical microscopy in a clinical setting.

## INTRODUCTION

Neoadjuvant radiochemotherapy has become a standard of care for patients with locally advanced rectal cancer. This preoperative treatment is associated with improved local control, reduced postoperative local recurrence, and higher sphincter preservation rate in rectal cancer patients and can improve postoperative survival and prognosis [1–3]. Studies have found that patients with a better response to preoperative treatment have a better prognosis [4, 5]. Thus, clinical outcome depends not only on the initial stage of the tumor, but also on the preoperative radiochemotherapy-induced tumor response [6].

However, treatment response varies among patients, ranging from a pathologic complete response to complete resistance. If a patient achieves a clinical complete response, surgery may be avoided entirely. However, most clinicians choose to perform resection in these patients due to the current limitations of medical imaging technology. Ideally, tumor response to neoadjuvant therapy should also be used to determine operation timing because, after neoadjuvant therapy, a tumor needs to progress from necrosis to apoptosis to complete fibrosis, but these pathologic changes cannot be assessed accurately in preoperative evaluation. Therefore, real-time monitoring of disease response to therapy requires standardization to design risk-adapted preoperative and postoperative treatments and ultimately optimize patient outcomes. However, assessment of neoadjuvant therapy response is currently suboptimal [7].

Fortunately, biological exploration is benefiting from continuing development of nonlinear optical imaging technique. This imaging technique is capable of high-resolution, high-penetration-depth, three-dimensional imaging of biological tissues and has the ability to detect cellular and subcellular tissue microstructure by excitation of intrinsic fluorescent molecules. As a result, in this study, we monitored therapy response to preoperative radiochemotherapy for rectal cancer using multimodal nonlinear imaging combining the second harmonic generation (SHG) and two-photon excited fluorescence (TPEF) signals.

## RESULTS

**Figure 1:**
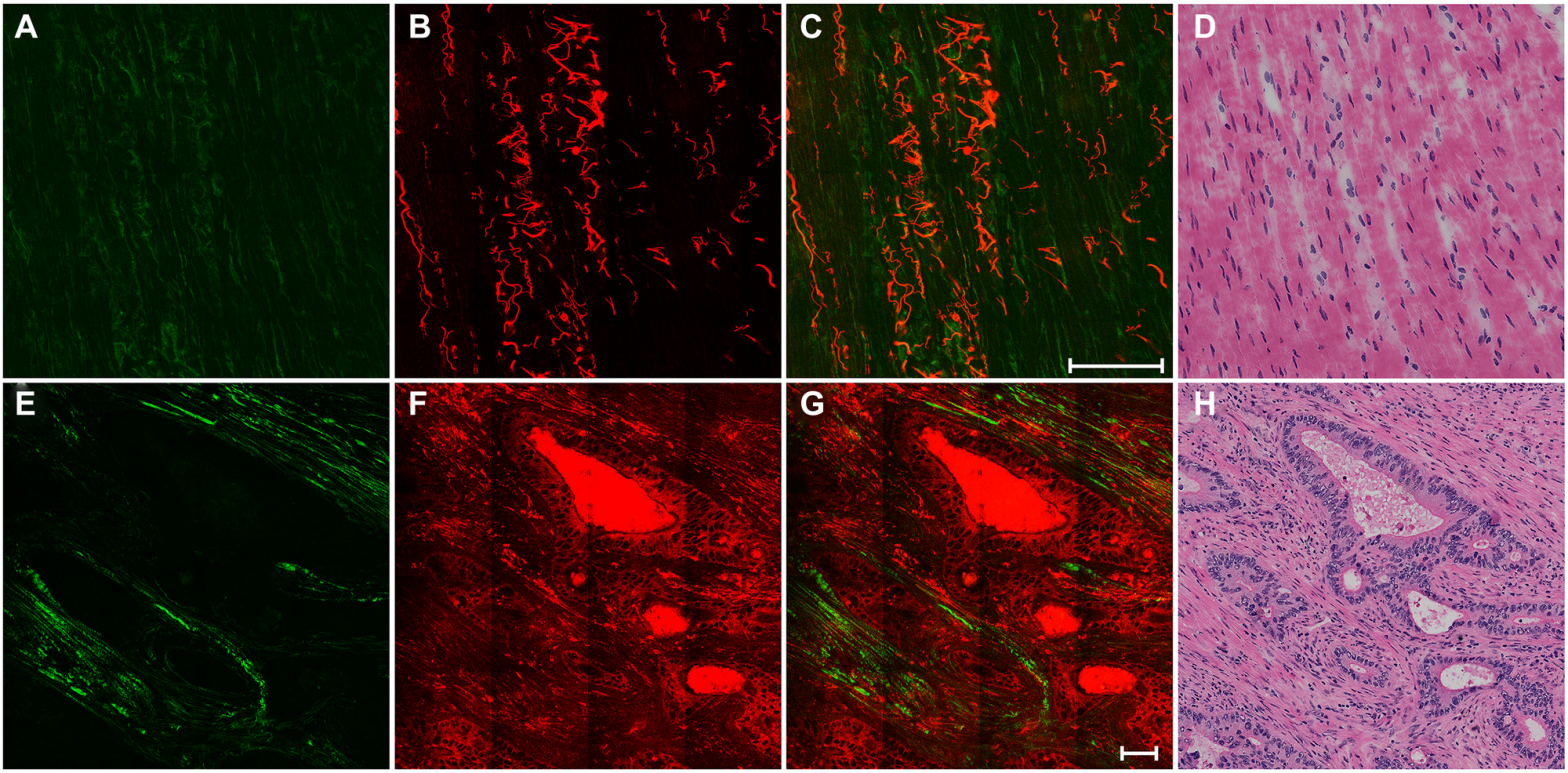
The top row shows the nonlinear optical images of normal muscularis and corresponding H&E-stained image (**A**) SHG image (color-coded green); (**B**) TPEF image (color-coded red); (**C**) merging of SHG and TPEF images; and (**D**) H&E image. The bottom row shows the nonlinear optical images of cancerous muscle tissue and corresponding H&E-stained image: (**E**) SHG image; (**F**) TPEF image; (**G**) merging of SHG and TPEF images; and (**H**) H&E-stained image. Scale bar: 100 μm.

The top row in Figure 1 shows representative SHG/TPEF images of normal muscularis propria and a corresponding hematoxylin and eosin (H&E)-stained image. Collagen fibers and elastic fibers in the normal muscular tissues can be easily distinguished. The collagen fibers are parallel to each other in Figure 1A through the SHG signal, whereas the elastic fibers have a long rope-like morphology in Figure 1B via the TPEF signal. This morphology allows spring-like coil and recoil. Overlay of the SHG and TPEF images is displayed in Figure 1C, and the detailed tissue microstructures shown in this image cannot be observed in the H&E-stained image shown in Figure 1D.

The bottom row in Figure 1 shows representative SHG/TPEF images of cancerous muscle tissues and a corresponding H&E-stained image. Compared with normal tissues, the striated muscular tissues are severely destroyed by tumor invasion and replaced by large amounts of cancerous glands. These glands are disordered and have an irregular shape, which is a typical feature of adenocarcinoma. A significant loss of the original collagen fibers and elastin fibers occurred; however, the invasion by tumor resulted in desmoplasia, and thus the glands are surrounded by collagen fibers (Figure 1E). These tissue architecture details correlate with the H&E-stained image (Figure 1H).

**Figure 2:**
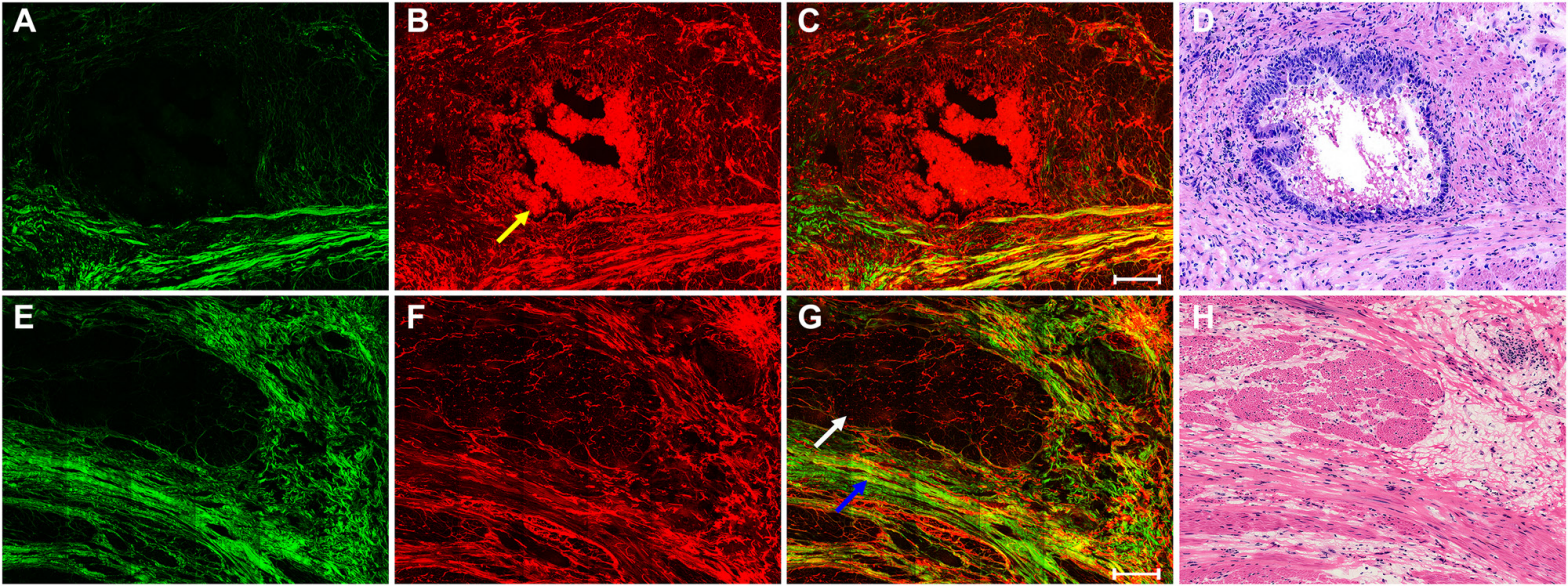
The top row shows the nonlinear optical images of tumor response to neoadjuvant therapy with fibrosis changes and corresponding H&E-stained image (**A**) SHG image; (**B**) TPEF image; (**C**) merging of SHG and TPEF images; and (**D**) H&E-stained image. The bottom row shows the nonlinear optical images of residual muscular tissues with predominant tumor regression and fibrosis after neoadjuvant therapy in rectal cancer and corresponding H&E-stained image: (**E**) SHG image; (**F**) TPEF image; (**G**) merging of SHG and TPEF images; and (**H**) H&E-stained image. White arrow: remaining muscle tissues; blue arrow: fibrosis; yellow arrow: dead cells. Scale bar: 100 μm.

The top row in Figure 2 displays representative SHG/TPEF images of tumor response to neoadjuvant therapy with fibrosis changes and a corresponding H&E-stained image. The SHG/TPEF images show that rectal tumors have regressed after preoperative radiochemotherapy and are gradually being replaced by collagen fibers and that large amounts of dead cells have accumulated in the gland lumen (yellow arrow in Figure 2B). As a result of fibrotic response induced by neoadjuvant therapy, the amount of collagen fibers has increased. In contrast to the collagen fibers in normal muscularis and desmoplastic response, the collagen fibers in fibrotic tissues are more disordered and show irregular crimping. These tissue microstructures cannot be observed directly from the H&E-stained image (Figure 2D).

The bottom row in Figure 2 shows representative SHG/TPEF images of residual muscular tissues with severe tumor regression and predominantly fibrotic changes after preoperative radiochemotherapy and a corresponding H&E-stained image. The rectal tumors have almost disappeared and are being replaced by fibrotic tissue (blue arrow in Figure 2G). Tumor regression after treatment in rectal carcinomas occurs mostly as fibrosis or fibroinflammatory changes that replace neoplastic glands [8, 9]. Only a small amount of residual muscular tissue remains [white arrow in (Figure 2G), which is interpreted as former tumor infiltration that led to the destroyed muscular layer. As shown in these images, multimodal nonlinear optical microscopy has many advantages compared with the H&E-stained image (Figure 2H)] and may be a valuable tool to investigate extracellular matrix remodeling after neoadjuvant therapy in rectal cancer.

**Figure 3:**
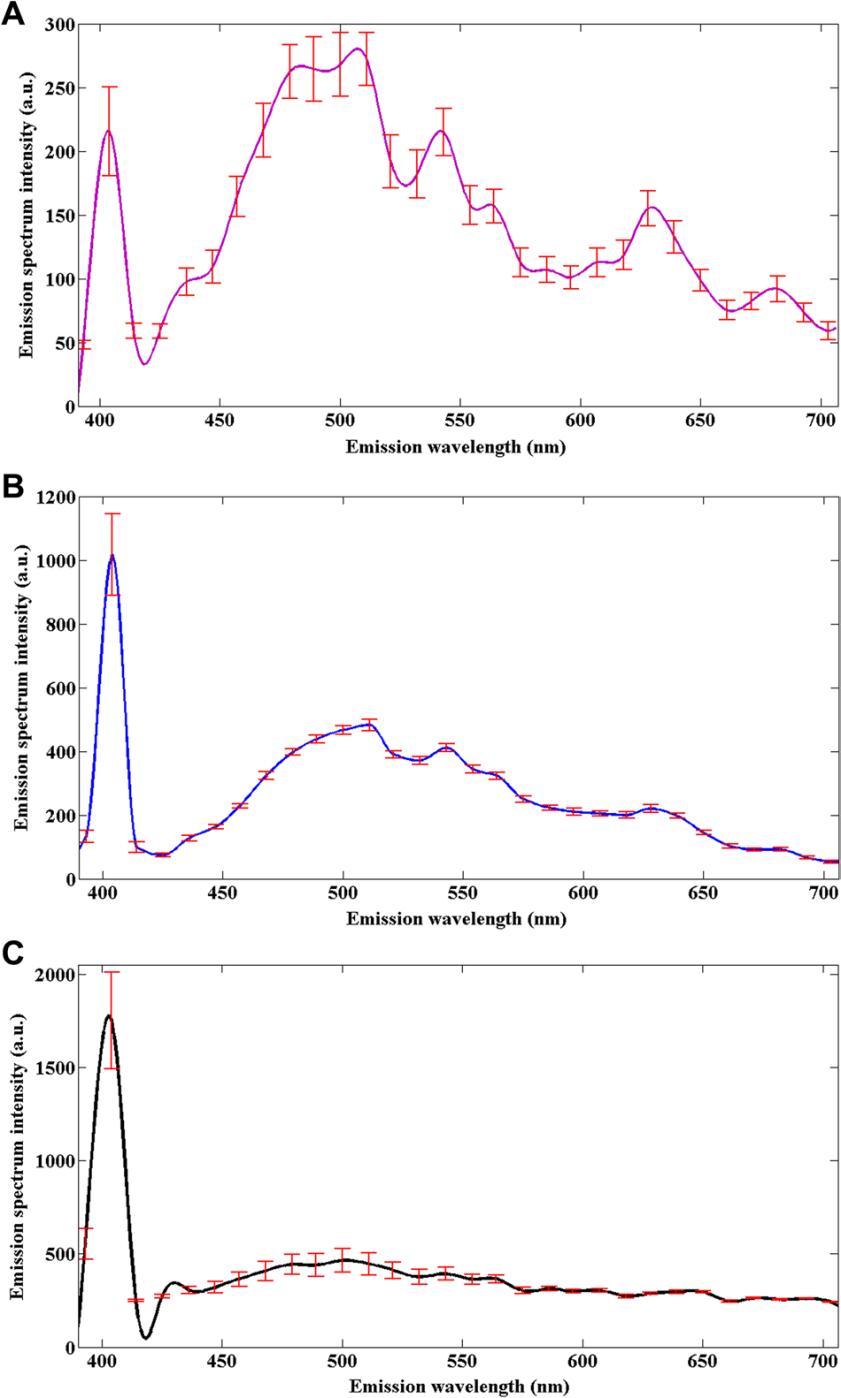
Nonlinear emission spectra of the normal muscular tissues (A), pretherapy muscular tissues (B), and posttherapy muscular tissues (C) in rectal cancer, obtained under the same conditions Error bars indicate standard deviation.

To determine the origin of nonlinear optical signals in normal muscular tissues and pretherapy and posttherapy muscular tissues in rectal cancer, emission spectroscopic investigations were performed using the spectral imaging scan mode under the same conditions. A total of 15 samples including five normal tissues, five pretherapy tissues, and five posttherapy tissues were selected for measuring the nonlinear emission spectra. The fresh tissue section of each sample was excited at an 810-nm excitation wavelength, and the emission signals were collected between 377 and 716 nm using a spectral detector under the lambda mode setting. As displayed in Figure 3, their spectra show obvious differences, but they all have two peaks at approximately 405 nm (half of the 810-nm excitation wavelength) and 511 nm. According to a previous publication, the 405-nm peak (SHG peak) originates from collagen, whereas the strong fluorescence peak at 511 nm (TPEF peak) corresponds to elastin [10].

**Figure 4:**
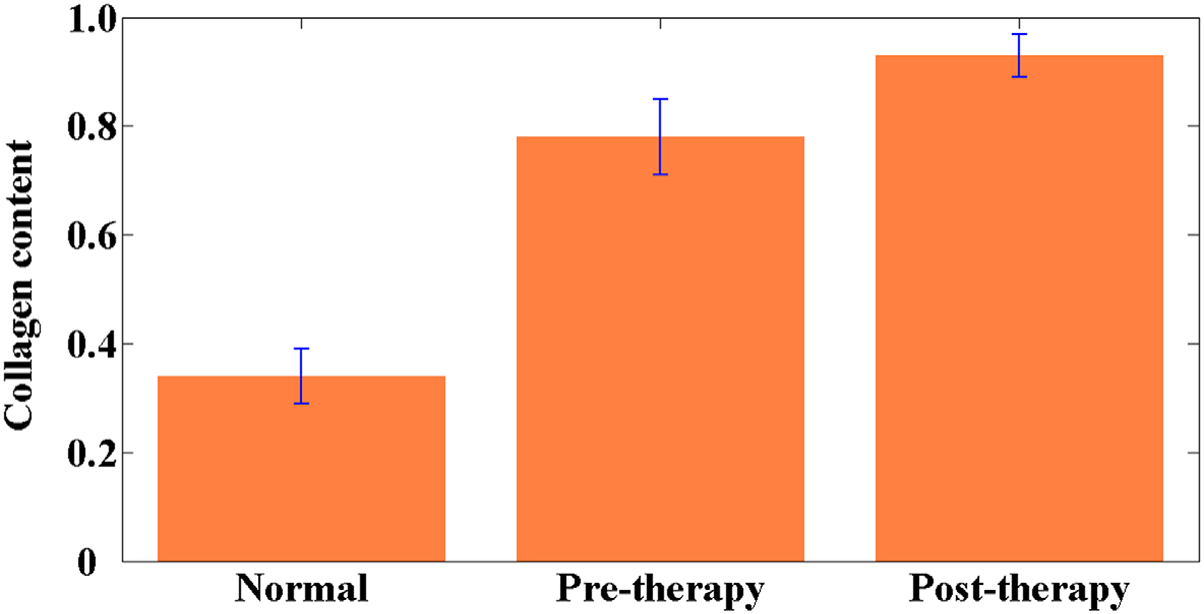
Collagen content in the normal, pretherapy, and posttherapy muscular tissues *P* < 0.001 was obtained between any two sample groups. Error bars indicate standard deviation.

Collagen content was also calculated to quantify stromal collagen changes. Quantitative results shown in Figure 4 reveal that collagen content in the normal muscular tissue was 0.34 ± 0.05, whereas in the pretherapy and posttherapy muscular tissues, collagen content was 0.78 ± 0.07 and 0.93 ± 0.04, respectively. Highly significant differences existed between any two sample groups (*P* < 0.001). These results indicate that the pretherapy and posttherapy muscular tissues may contain more collagen fibers because of the desmoplastic response caused by tumor invasion and the fibrotic response induced by neoadjuvant therapy, respectively. Therefore, collagen content may be treated as an optical biomarker to discriminate between normal and abnormal tissues.

**Figure 5:**
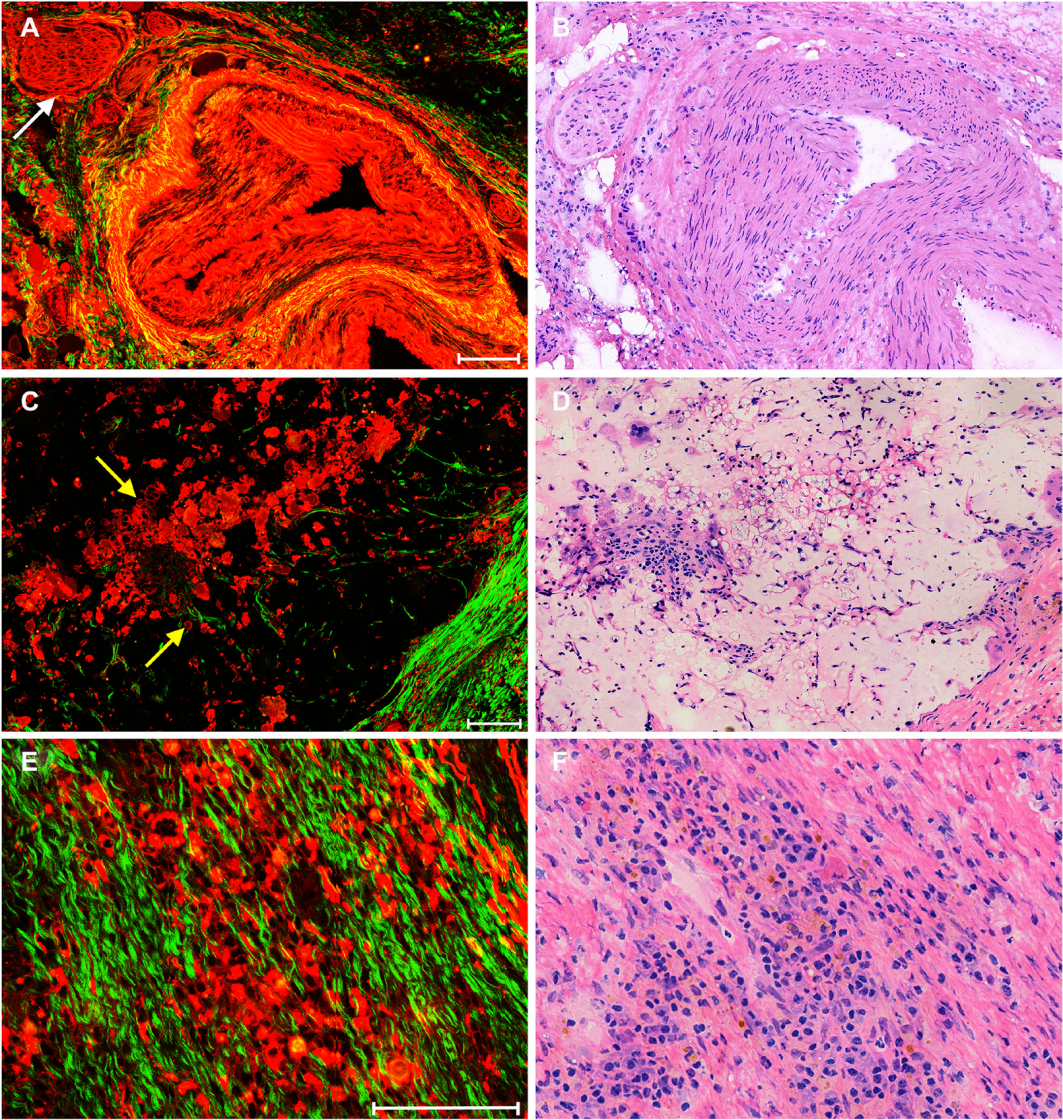
Representative nonlinear optical images of stromal response after neoadjuvant therapy in rectal carcinoma and corresponding H&E-stained images including (**A**) merging of SHG and TPEF images of blood vessel hyperplasia and (**B**) corresponding H&E-stained image; (**C**) merging of SHG and TPEF images of colloid response and (**D**) corresponding H&E-stained image; and (**E**) merging of SHG and TPEF images of inflammatory reaction and (**F**) corresponding H&E-stained image. White arrow: nerve fibers; yellow arrows: tumor cells. Scale bar: 100 μm.

Figure 5 displays representative SHG/TPEF images of stromal response after neoadjuvant therapy in rectal carcinoma and corresponding H&E-stained images. Because elastin in blood vessels can generate strong TPEF signal, changes in blood vessels, such as hyperplasia, are easily found. The blood vessel in (Figure 5A) shows severe thickening, and its lumen has almost disappeared, which may result in bowel ischemia or infarction [11, 12]. To our surprise, nerve fibers [white arrow in (Figure 5A)] are also detected through TPEF signal.

Colloid response, which is a type of stromal response and is often seen in posttreatment rectal tumors, is defined by predominant colloid changes with or without residual tumor cells and is an important predictor of survival [13]. In our study, colloid response can be directly identified in the nonlinear optical image (Figure 5C), where mucin is black in color and residual tumor cells [yellow arrow in (Figure 5C)] float in mucin lakes. Inflammatory cell infiltration is also of major interest because it has long been regarded as a type of host response and an important factor in tumor regression [8, 14]. Figure 5E reveals that large amounts of inflammatory cells have infiltrated into fibrotic-type stroma, indicating that an inflammatory reaction occurs in patients after neoadjuvant radiochemotherapy. These details are in agreement with the corresponding H&E-stained images of the paired histologic sections (Figures 5B, 5D, and 5F).

Previous studies have demonstrated that stromal fibrosis induced by preoperative adjuvant treatment is an important indicator of wound healing [15, 16] and that the type of fibrotic response is associated with prognosis in rectal cancer [17, 18]. Fibrous stroma is pathologically classified into three patterns: mature (layered collagen), intermediate (keloid-like collagen), and immature (myxoid stroma). Five-year disease-free survival rates were 82%, 72%, and 47% for mature, intermediate, and immature stroma, respectively [19]. Thus, identification of stromal pattern has clinical significance because it can be used as a potential histoprognostic marker for decisions on postoperative treatment.

**Figure 6:**
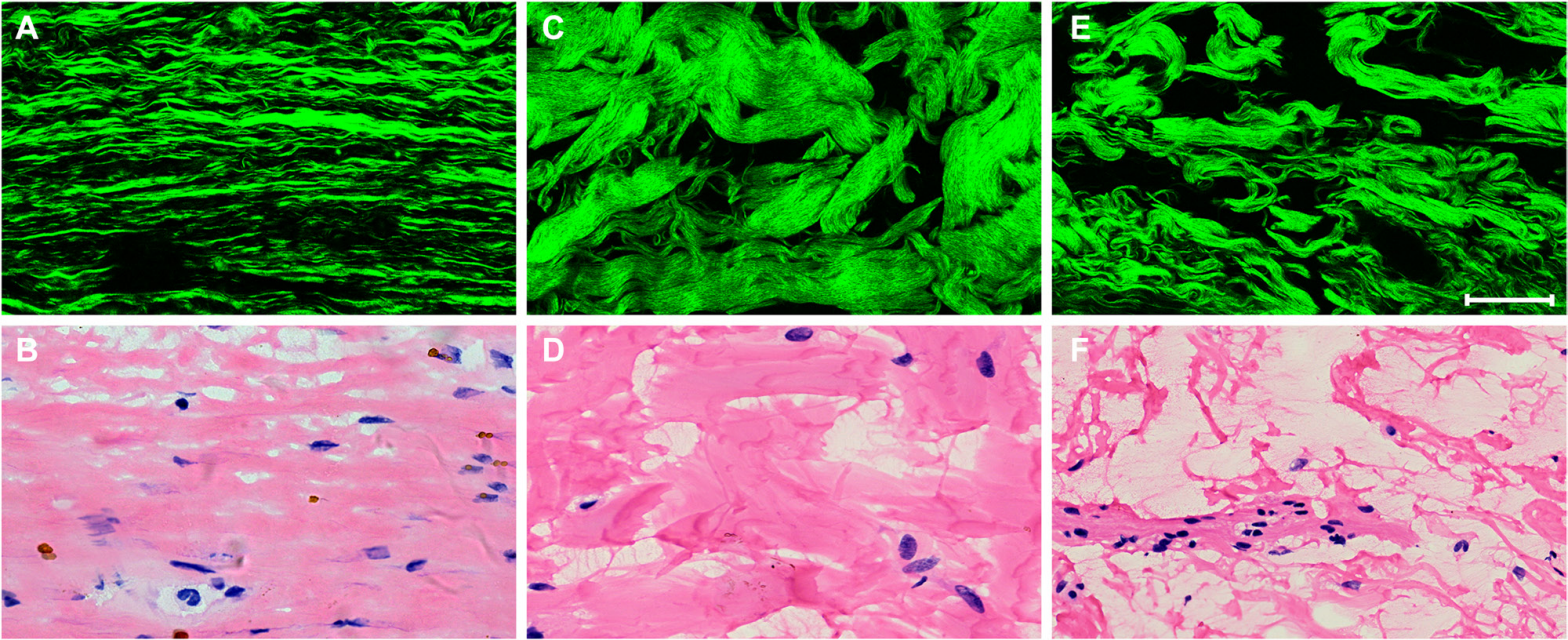
Representative nonlinear optical images of three types of fibrotic response after neoadjuvant therapy in rectal carcinoma and corresponding H&E-stained images (**A**) SHG image of mature fibrotic stroma and (**B**) corresponding H&E-stained image; (**C**) SHG image of intermediate fibrotic stroma and (**D**) corresponding H&E-stained image; (**E**) SHG image of immature fibrotic stroma and (**F**) corresponding H&E-stained image. Scale bar: 50 μm.

Figure 6 shows SHG images of different types of fibrous stroma after neoadjuvant therapy in rectal carcinoma and corresponding H&E-stained images. Our data suggest that collagen fibers are elongated and stratified into multilayers in mature stroma (Figure 6A), are keloid-like in intermediate stroma (Figure 6C), and are disordered and intermingled with mucus in immature stroma (Figure 6E). These tissue microarchitectural characteristics obtained from SHG microscopy are consistent with the corresponding H&E-stained images (Figures 6B, 6D, and 6F).

**Figure 7:**
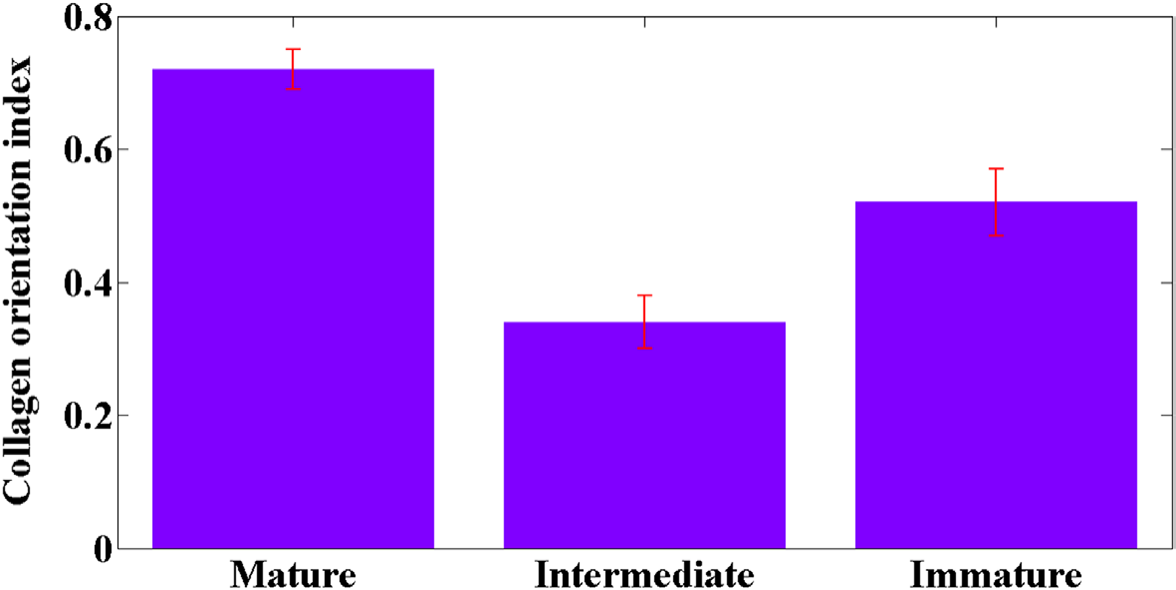
Collagen orientation index in mature, intermediate, and immature stroma *P* < 0.001 was obtained between any two sample groups. Error bars indicate standard deviation.

Compared with the intermediate and immature stroma, the most obvious feature of collagen fibers in mature stroma is that they are fine and well-organized. Therefore, measurement of the orientation of collagen fibers may provide better understanding of patterns of fibrosis for pathologists. As previously reported, the collagen orientation index ranges from 0 (perfectly random) to 1 (perfectly parallel) and is measured by ImageJ software [20, 21]. In contrast to immature stroma (0.52 ± 0.05) and intermediate stroma (0.34 ± 0 .04), the collagen orientation index in mature stroma (0.72 ± 0.03) is increased, as shown in Figure 7, indicating that collagen fibers become more ordered in mature stroma. In addition, there are highly significant differences (*P* < 0.001) in the collagen orientation index between the three types of stroma. Hence, collagen orientation index can be used as a diagnostic parameter to distinguish between the three kinds of fibrous stroma.

## DISCUSSION

Neoadjuvant radiochemotherapy has been proven to be a useful tool for tumor reduction and to increase operability and decrease the rate of local recurrence in colorectal cancer patients [22, 23]. However, no effective method has existed for directly monitoring the effects of preoperative radiochemotherapy or the treatment dose and regimen. Fortunately, preoperative adjuvant treatment modifies the histologic appearance of rectal carcinoma, mainly through tumor regression and stromal changes, which are clinically meaningful markers because they have been correlated with outcome [8, 11]. These histopathologic changes make it possible to monitor therapy response by nonlinear optical methods because of the intrinsic optical signals in biological tissues [24].

**Figure 8:**
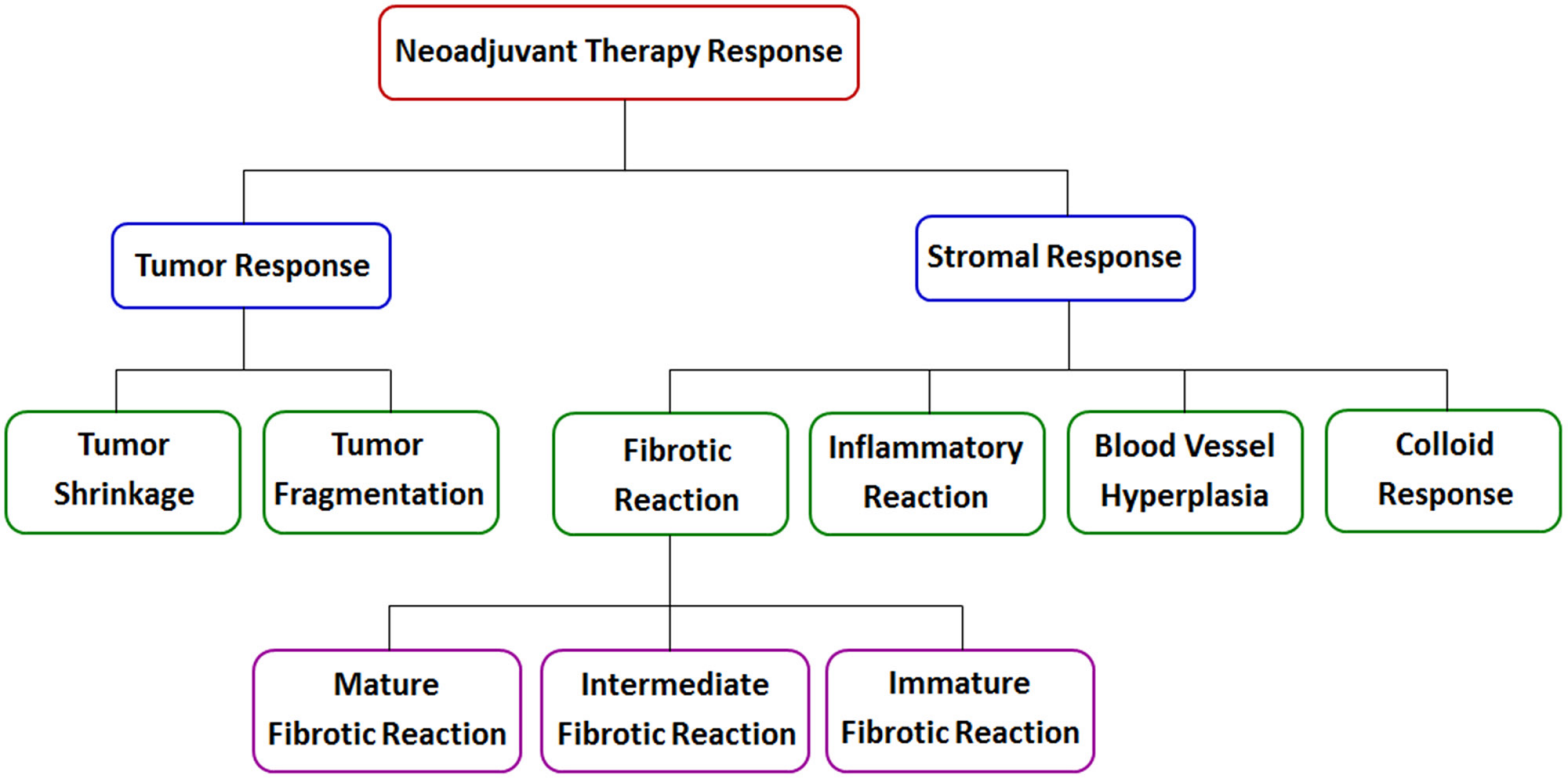
Schematic representation of neoadjuvant therapy response in rectal cancer

**Figure 9:**
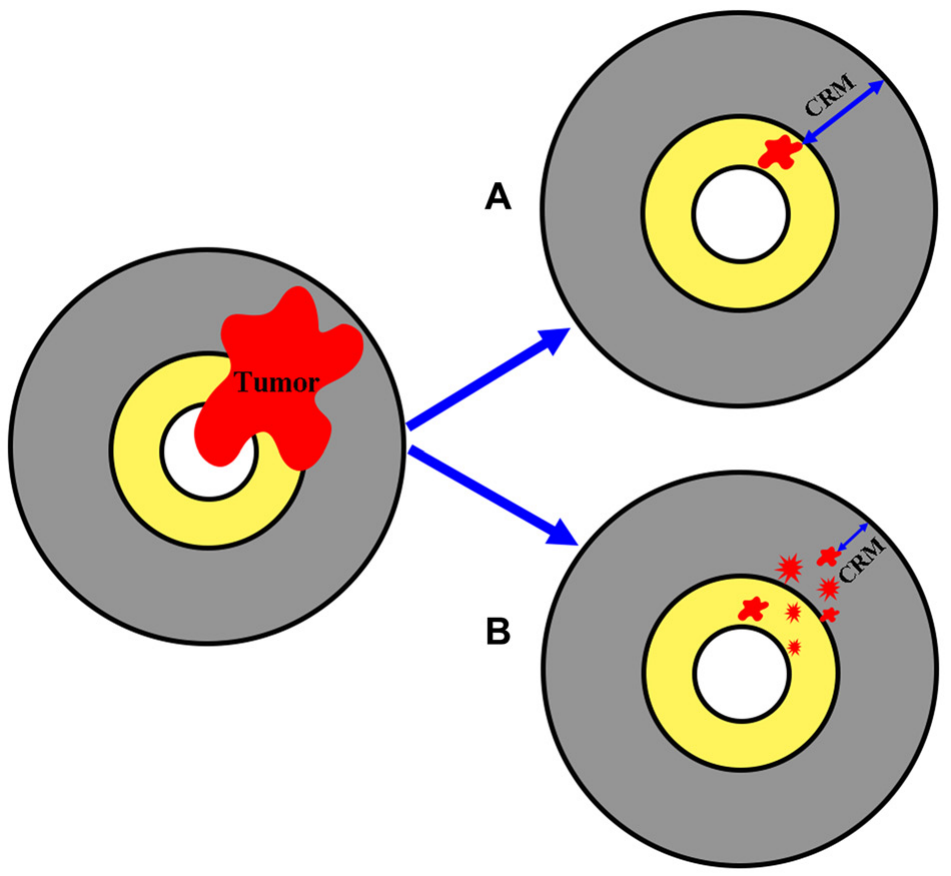
Schematic representation of two possible forms of tumor response to neoadjuvant therapy (**A**) tumor shrinkage and (**B**) tumor fragmentation.

Therapy response in rectal cancer patients treated with preoperative radiochemotherapy is divided into two main groups: tumor response and stromal response, as shown in Figure 8. Tumor regression is important because the chance of obtaining a negative circumferential resection margin (CRM) is increased after extensive tumor regression; however, tumor response can take the form of either tumor shrinkage or tumor fragmentation [25]. Tumor shrinkage reflects good response and entails a favorable prognosis because it is associated with a larger negative CRM, as displayed in Figure 9A. In contrast, tumor fragmentation is more likely to have poor prognostic characteristics because it generates mesorectal tumor deposits or tumor scatter, decreasing the size of the negative CRM, as shown in Figure 9B.

The scenario of tumor fragmentation indicates that the degree of tumor regression is not informative for depth of infiltration because residual tumor cells may still be scattered throughout the rectal wall or mesorectum, and a report has indicated that normal-appearing mucosa may harbor microscopic tumor cells up to 3 to 4 cm in any direction [26]. However, due to the limited resolution of medical imaging technology [27], detecting tumor cell deposits and tumor scatter is a challenge, and failure to do so can result in incomplete resection and a poor prognosis. In addition, assessing tumor regression, which is based on the tumor regression grading system [15, 28], does not accurately distinguish tumor fragmentation from tumor shrinkage [29].

Our experimental data imply that without the use of any exogenous contrast agents, TPEF microscopy can directly identify a single tumor cell because of its subcellular-level resolution. In addition, as a continuing development of nonlinear microscopy, it can also scan a large area of the samples [30]. Therefore, nonlinear microscopy may help to distinguish between tumor shrinkage and fragmentation and to identify tumor deposits or tumor scatter, which will help clinicians determine the surgical margin during operation.

There are many diverse elements in tumor stroma, and these have traditionally been classified into three main categories: fibrous tissues, blood vessels, and inflammatory cells. Accordingly, stromal changes induced by preoperative radiochemotherapy mainly include fibrotic reaction, inflammatory reaction, blood vessel hyperplasia, colloid response, and so on. Our study shows that TPEF microscopy is able to monitor inflammatory cell infiltrates, vascular proliferation, and colloid reaction without the need for fluorescent markers because there are several endogenous fluorophores within rectal tissues, including nicotinamide adenine dinucleotide (NADH) and flavin adenine dinucleotide (FAD) in cells and elastin in the blood vessel wall. Using traditional techniques, it is difficult to detect sparse residual tumor cells in mucin lakes on the resection margin, especially in frozen sections [7], which may lead to unnecessary surgical extensions [11, 13]. However, our study demonstrates that TPEF imaging can detect the rare residual carcinomatous cells floating in mucin pools because tumor cells generate TPEF signals, whereas mucin does not.

Tumor invasion may activate fibroblasts and cause a desmoplastic reaction, whereas tumor regression in posttreatment rectal carcinomas mostly results in fibrotic changes replacing neoplastic glands [17–19]. As previously noted, fibrous response is grouped into mature, intermediate, and immature types, and better understanding of patterns of fibrosis might result in prognostic factors that could be useful in designing risk-adapted postoperative therapies for patients. Although these fibrotic changes can be determined by histopathologic examination in surgically resected specimens, the procedure is troublesome and time consuming, and diagnostic results can only be acquired postoperatively. In this study, our data indicate that SHG imaging can be valuable for studying fibrotic pathologies. SHG microscopy not only can discriminate between fibrotic patterns, but also can provide quantitative information, including the collagen content and collagen orientation. Thus, SHG microscopy can be used to help determine operation timing by assessing the degree of fibrosis after preoperative radiochemotherapy in rectal cancer.

To summarize, nonlinear optical microscopic imaging possesses many unique advantages, such as label-free imaging, single-cell detection, high-penetration-depth, and reduced photobleaching and phototoxicity in the out-of-focus regions. As a result, it has been widely used in life sciences [31]. Although our study was conducted using *ex vivo* samples, it demonstrated great potential for future clinical applications when *in vivo* nonlinear optical endoscopy is realized, especially for presurgery evaluation after neoadjuvant radiotherapy. As a novel optical tool, the exploitation of a compact and flexible nonlinear optical endoscope for *in vivo* imaging is attracting attention and research, and previous publications have shown that *in vivo* images of unstained colon, kidney, and liver from an anesthetized rat can be obtained using a compact and flexible multiphoton microendoscope [32, 33]. Thus, once the nonlinear optical endoscope is capable of serving as an advanced endoscopic imaging technology for clinical examinations, our work may provide the groundwork for the application of this endoscopy in the monitoring of neoadjuvant therapy response in rectal cancer.

## CONCLUSIONS

In summary, it is presented that multimodal nonlinear optical microscopy can be used to monitor therapy response to neoadjuvant treatment. Our results demonstrate that a combination of SHG and TPEF imaging can identify pathologic alterations such as tumor response and stromal response in rectal cancer patients treated with preoperative radiochemotherapy. In the near future, with further improvements in these nonlinear methods, this ability may impact treatment options in rectal cancer patients who have received preoperative radiochemotherapy because the effects of therapy can be assessed before surgery. When nonlinear microscopy develops into a real-time, label-free, *in vivo* imaging technique, it will help clinicians to alter treatment doses if needed, determine the operation plan and operation timing, and avoid overtreatment or forgo futile treatment.

## MATERIALS AND METHODS

### Nonlinear optical imaging system

The nonlinear optical imaging system used in this study has been previously described in detail [34, 35]. In short, an inverted microscope (LSM 510 META; Zeiss, Oberkochen, Germany) equipped with a mode-locked femtosecond Ti:sapphire laser (Mira 900-F; Coherent, Inc., Santa Clara, CA) was used to obtain high-resolution images. An oil immersion objective (Plan-Apochromat 63×, NA=1.4; Zeiss) was used for focusing the excitation beam into samples and also for collecting the backscattered intrinsic SHG and TPEF signals. The META detector with eight independent channels in this system consists of a reflective grating and an optimized 32-channel PMT array detector to collect emission signals within the random range from 377 to 716 nm. In this study, two independent channels were chosen to collect SHG and TPEF signals, where one channel covered the wavelength range from 389 to 419 nm for collection of SHG signal and another channel covered the wavelength range from 430 to 716 nm for collection of TPEF signal at an excitation wavelength of 810 nm. To increase the contrast of SHG/TPEF images, the SHG image was color coded in green and the TPEF image was color coded in red.

### Sample preparation

This study was conducted with the approval of the institutional review board at the Fujian Medical University Union Hospital, and signed informed consent was obtained from each patient. Posttherapy tumors penetrating into the muscular layer or deeper were selected for this study, and normal tissues and pretherapy cancerous tissues were also collected for comparison. The region of our interest was muscular tissues because once tumor infiltrates into or beyond the muscularis propria layer, it is classified as an advanced cancer and has a much worse prognosis.

Thirty-two patients with adenocarcinoma of rectum who received neoadjuvant therapy were recruited. Patient age ranged from 38 to 83 years (59 ± 11 years), and the male-to-female ratio was 1.7. Once fresh tissue samples were removed by surgeons, they were sent to the pathology laboratory immediately, where each specimen was serially sectioned at 10 μm by cryostat microtome. Then five serial tissue slices were chosen for research; the middle slice was stained with H&E to confirm experimental results, and the other sections were used for nonlinear optical imaging. In addition, to avoid dehydration or shrinkage during the imaging process, a small amount of phosphate-buffered saline was applied to the specimen.

### Histologic analysis

All of the H&E-stained slices were reviewed by a certified pathologist, and images were then taken using a standard bright field light microscope (Eclipse Ci-L; Nikon Instruments Inc., Tokyo, Japan) with a CCD (DS-Fi2; Nikon). The results from nonlinear optical imaging were then compared with the H&E images obtained from light microscopy (40×) for confirmation.

### Statistical analysis

To quantitatively assess collagen changes in stroma during neoadjuvant therapy, collagen content and collagen orientation index were measured, where collagen content was defined as the ratio of SHG to all pixels in each image and collagen orientation index was described by an index ranging from 0 (perfectly random) to 1 (perfectly parallel). These values were expressed as means and standard deviations. Statistical calculations were performed using the IBM SPSS Statistics 21, and *P* values of ≤0.05 were considered statistically significant.
